# Chitosan/Sodium Dodecyl Sulfate Complexes for Microencapsulation of Vitamin E and Its Release Profile—Understanding the Effect of Anionic Surfactant

**DOI:** 10.3390/ph15010054

**Published:** 2021-12-31

**Authors:** Jelena Milinković Budinčić, Lidija Petrović, Ljiljana Đekić, Milijana Aleksić, Jadranka Fraj, Senka Popović, Sandra Bučko, Jaroslav Katona, Ljiljana Spasojević, Jelena Škrbić, Anđelija Malenović

**Affiliations:** 1 Department of Biotechnology and Pharmaceutical Engineering, Faculty of Technology Novi Sad, University of Novi Sad, Bulevar Cara Lazara 1, 21000 Novi Sad, Serbia; lidijap@uns.ac.rs (L.P.); maja.maki16@gmail.com (M.A.); jadrankam@gmail.com (J.F.); 2 Department of Pharmaceutical Technology and Cosmetology, Faculty of Pharmacy, University of Belgrade, 11221 Belgrade, Serbia; ljiljanadjek@gmail.com; 3 Department of Food Preservation Engineering, Faculty of Technology Novi Sad, University of Novi Sad, Bulevar Cara Lazara 1, 21000 Novi Sad, Serbia; madjarev@uns.ac.rs; 4 Department of Applied and Engineering Chemistry, Faculty of Technology Novi Sad, University of Novi Sad, Bulevar Cara Lazara 1, 21000 Novi Sad, Serbia; sandranj@uns.ac.rs (S.B.); jkatona@uns.ac.rs (J.K.); lj.spasojevic@tf.uns.ac.rs (L.S.); jelenaskrbic1994@gmail.com (J.Š.); 5 Department of Drug Analysis, Faculty of Pharmacy, University of Belgrade, 11221 Belgrade, Serbia; andja@pharmacy.bg.ac.rs

**Keywords:** chitosan, sodium dodecyl sulfate, vitamin E, microencapsulation, spray drying in vitro release kinetics

## Abstract

Microencapsulation of bioactive substances is a common strategy for their protection and release rate control. The use of chitosan (Ch) is particularly promising due to its abundance, biocompatibility, and interaction with anionic surfactants to form complexes of different characteristics with relevance for use in microcapsule wall design. In this study, Ch/sodium dodecyl sulfate (SDS) microcapsules, without and with cross-linking agent (formaldehyde (FA) or glutaraldehyde (GA)), were obtained by the spray drying of vitamin E loaded oil-in-water emulsion. All of the microcapsules had good stability during the drying process. Depending on the composition, their product yield, moisture content, and encapsulation efficiency varied between 11–34%, 1.14–1.62%, and 94–126%, respectively. SEM and FTIR analysis results indicate that SDS as well as cross-linkers significantly affected the microcapsule wall properties. The profiles of in vitro vitamin E release from the investigated microcapsules fit with the Korsmeyer-Peppas model (r^2^ > 0.9). The chemical structure of the anionic surfactant was found to have a significant effect on the vitamin E release mechanism. Ch/SDS coacervates may build a microcapsule wall without toxic crosslinkers. This enabled the combined diffusion/swelling based release mechanism of the encapsulated lipophilic substance, which can be considered favorable for utilization in food and pharmaceutical products.

## 1. Introduction

Vitamin E, as a natural antioxidant that delays photo aging and provides protection of tissues from UV radiation as well as a moisturizing effect, nowadays has an important place in the production of cosmetics, drugs, and food. Vitamin E is accepted in Europe as a food additive and thus occurs in many food substances that are consumed as part of the normal diet [1].α-Tocopherol is widely used as a pharmaceutical excipient [2]. It belongs to the most commonly used antioxidants in pharmaceutical preparations for oral, topical, and parenteral administration and it is included in the FDA Inactive Ingredients Database [3]. Moreover, it is one of the most used active substances of natural origin, even though it is often unstable [4,5,6]. However, utilization of its beneficial effects is limited due to its instability to high temperature, oxygen, and light. For these reasons, vitamin E (α-tocopherol) cannot be used directly in functional products without the risk of reducing its bioactivity, i.e., microencapsulation is needed for its protection during storage [6,7,8,9,10].

Microencapsulation is a process in which bioactive compounds in solid, liquid or gas state are protected against environmental harsh conditions by a coating wall. Several microencapsulation techniques are available for use, and their selection should be done according to the bioactive material to be encapsulated and the particles’ final purpose [7,8,11]. Microencapsulation by spray drying is an economic, rapid, simple, reproducible, continuous, and efficient technique, which is mostly used for the encapsulation of bioactive compounds, such as polyphenols, enzymes, vitamins, and its derivatives.

The porosity and mechanical resistance of the microcapsule wall are significantly affected by the nature and amount of added cross-linking agent. This influences the release kinetics of encapsulated active substances, as well as its protection from environmental factors [12]. Regarding food, cosmetic, and pharmaceutical applications, the wall material should be non-toxic and biocompatible [13,14]. One of them is chitosan (Ch), which is the commonly used polymer in these industries. It is a natural, non-toxic, biodegradable, and biocompatible substance with antibacterial properties, heavy metal ion chelation ability, and gel-forming properties.

Ch is obtained by the chemical derivatization of many different sea crustaceans, such as crabs or shrimps. It is a linear polysaccharide composed of D-glucosamine (deacetylated unit) and distributed β-(1-4)-linked N-acetyl-D-glucosamine (acetylated unit) [15,16,17,18,19]. The most common cross-linking agents used with chitosan are dialdehydes [16], which imply covalent binding [20]. However, the main disadvantage for the use of these kinds of cross-linking agents is their toxicity [21]. Moreover, pure Ch has a weak surface activity in aqueous solutions, similar to most of the strong polyelectrolytes, which make its use as a wall microcapsule material challenging. Anionic surfactants, such as sodium lauryl ether sulfate (SLES) and sodium dodecyl sulfate (SDS), show a significant reduction in surface tension at very low concentrations due to the strong adsorption of molecules at the liquid-air surface, which is typical for most of the surfactants in water solutions [22,23]. Previous studies have shown that chitosan can interact with anionic surfactants, and thus forms chitosan/surfactant complexes of different structures and properties [18,22,23,24]. This phenomenon is known as complex coacervation and is considered as the spontaneous liquid/liquid phase separation in colloidal systems given by the electrostatic interaction between two oppositely charged polymers and surfactant head groups [25,26,27]. Some surface active complexes may be used as emulsion stabilizers and emulsions could be converted into microcapsules suitable for food and pharmaceutical applications [17,22,23,24,28,29,30,31]. SDS is a common, synthetic anionic surfactant and it is structurally comparable to numerous biosurfactants that are widely used in many products. Primarily, it is used in the pharmaceutical and food industries, unlike SLES, which is less irritating and susceptible to degradation, and has a current widespread use in cosmetic formulations [32,33]. SLES differs from SDS due to the presence of ethoxyl groups between the sulphate polar head and the alkyl chain, which may lead to different mechanisms of interaction with Ch [34].

Our previous study evaluates Ch/SLES complexes as the wall material of oil content microcapsules enriched with vitamin E and their properties [5]. The study proved that the Ch/SLES complex can be used as a wall material for microencapsulation without the addition of toxic cross-linking agents, such as formaldehyde (FA) and glutaraldehyde (GA). However, as far as we know, vitamin E encapsulation in microcapsules based on Ch/SDS complexes has not been investigated to date. In addition, the potential distinctions in the properties of microcapsules related with the chemical structure differences between SLES and SDS have not been elucidated yet.

Therefore, the present study aims to microencapsulate vitamin E (α-tocopherol) by applying the spray drying technique and to evaluate Ch/SDS complexes as wall materials. The obtained microcapsules were subsequently characterized in terms of product yield, moisture content, particle size, and size distribution and morphology.Moreover, chemical interactions between functional groups of the complex constituents were investigated, the encapsulation efficiency was determined, and the kinetics and mechanism of vitamin E release from the Ch/SDS-based microcapsules were analyzed.

## 2. Results and Discussion

Microcapsules enriched with vitamin E were obtained by spray drying, using Ch and SDS in mass ratio of 1:2 as a wall material, with or withoutcross-linking agent. Thereafter, regarding all of the microcapsules, the yield was determined and the moisture content was characterized by the particle size and surface morphology.

### 2.1. Yield Analysis and Moisture Content of Microcapsules

**Figure 1:**
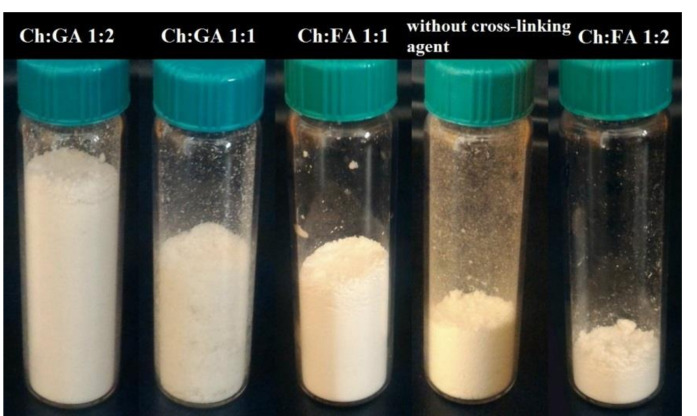
Yield of microcapsules obtained by the spray drying of 100 g of each microcapsule suspension sample.

The microcapsule production yield shows how much powder mass is produced after the spray drying process. In addition, it is expressed by the percentage in regards to the core and wall materials present in the initial emulsion. The yield of the process varies from 34%, which was achieved for the microcapsules cross-linked with GA in mass ratio Ch:GA 1:2, via the microcapsules cross-linked with GA and FA in mass ratio Ch:GA (and Ch:FA) 1:1, where the yield was around 21% and 15%, respectively, and microcapsules withoutcross-linking agent (13%), to a minimum yield of 11%, which was obtained for microcapsules cross-linked with FA in mass ratio Ch:FA 1:2 (Figure 1).

The relatively poor values of the yield were probably caused by the powder adherence on the interior surface of the drying chamber due to the high adhesive ability of Ch [35].

The moisture content is an important variable related to the shelf life of powders and often conducted to check on the spray-drying process. It was defined as the weight percentage of water in relation to the dry weight. Table 1 shows the mean value and the standard deviation of moisture content of the investigated microcapsules.

Taking into account the moisture content of the microcapsules shown in Table 1, from 1.14 to 1.62%, its values are in accordance with the results from other studies [36,37] and does not indicate a significant difference between the microcapsules including our study that refers tomicrocapsules with the Ch/SLES complex [5]. The spray drying process proved to be well optimized for obtaining vitamin E microcapsules and this low value for moisture content may contribute to the powder stability during the storage and prevention of changes in physical and chemical characteristics.

### 2.2. Encapsulation Efficiency 

The encapsulation efficiency was determined using UV-VIS spectrophotometry after extraction of vitamin E from microcapsules with 80% ethanol [38]. In addition, it was expressed as the percentage of encapsulated vitamin E relative to the total used in the process of emulsion preparation. The results are shown in Table 2.

Based on Table 2, significant differences were obtained between the encapsulation efficiency of microcapsules without cross-linking agent and cross-linked microcapsules. Therefore, the encapsulation efficiency was the highest in microcapsules without cross-linking agent and lowest in microcapsules cross-linked with GA in mass ratio 1:2. This result for microcapsules without cross-linking agent was expected given the small droplet size and narrower distribution. A slightly higher encapsulation efficiency of 100% for all of the other samples, except for microcapsules cross-linked with GA in mass ratio 1:2, can be explained by the fact that larger drops burst under given conditions during the drying process and the oil content was released together with vitamin E, which was adsorbed on the surface of the microcapsules. This observation coincides with the yield of microcapsules after production (Figure 1), which was the highest for the microcapsules cross-linked with GA in mass ratio 1:2 as a consequence of the formation of a more compact wall of microcapsule. The addition of cross-linking agent in Ch:GA (and Ch:FA) at mass ratio 1:1 did not significantly affect the encapsulation efficiency.

### 2.3. Droplet/Particle Size Distribution

**Figure 2:**
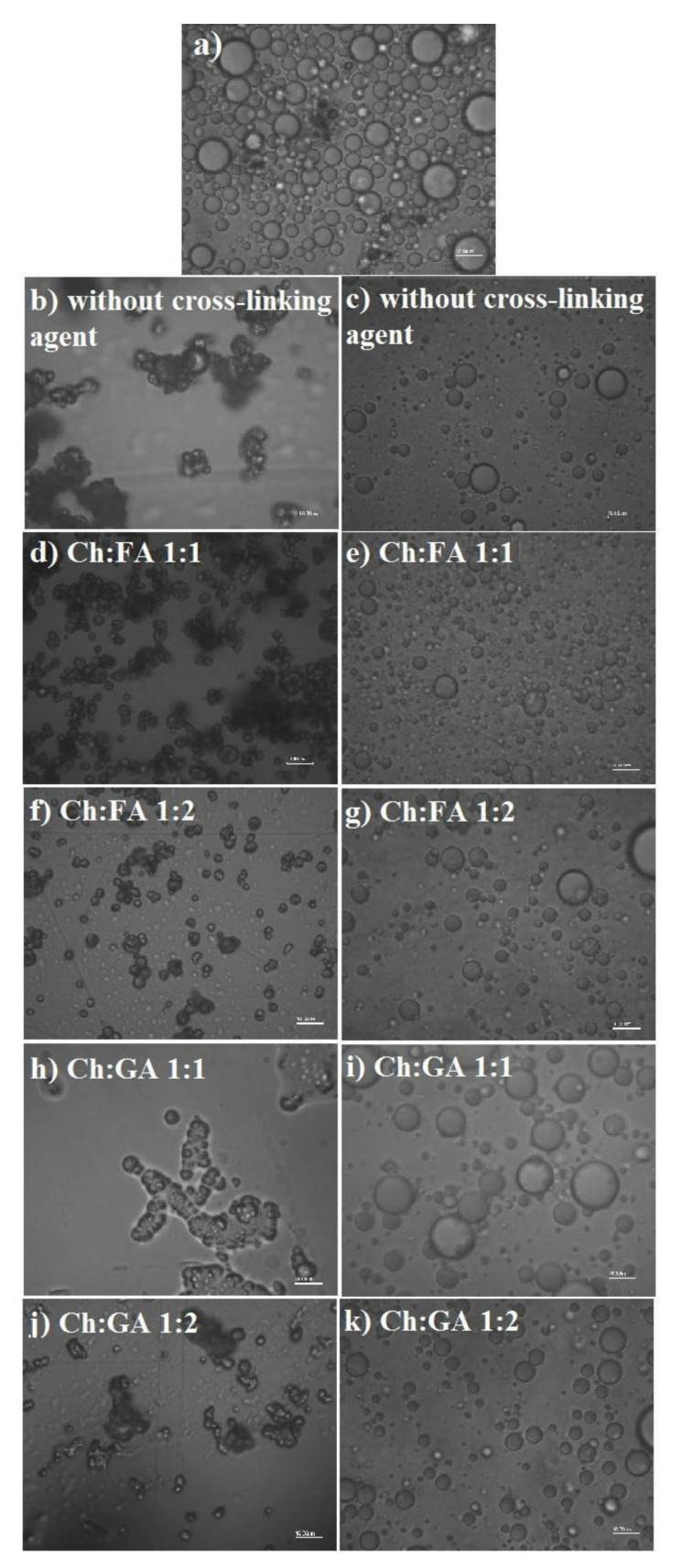
Microphotographs of: (**a**) Emulsion before drying; (**b**,**d**,**f**,**h**,**j**) chitosan/sodium dodecyl sulfate (Ch/SDS) microcapsules powders; (**c**,**e**,**g**,**i**,**k**) suspensions of the microcapsules in water.

Figure 2 shows microphotographs of emulsion before spray draying (a), microcapsules powders, and their suspensions in water obtained at different Ch:cross-linking agent ratios and without cross-linking agent.

All of the obtained microcapsules were stable and well re-dispersed in water. The photomicrographs of microcapsule powders (Figure 2b,d,f,h,j) show the absence of oil drops around them, indicating the high stability of the adsorption layer during the drying process.

Software Belview7 was used to process the microphotographs of emulsions and microcapsule suspensions re-dispersed in water. The distribution parameters, mean particle diameters, and standard deviationare shown in Table 3.

**Figure 3:**
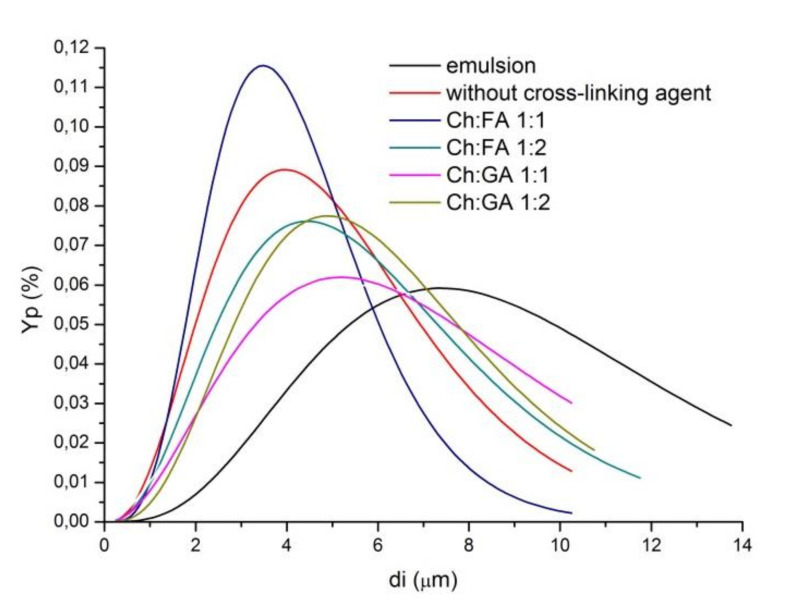
Droplet and particle size distribution of the initial emulsion and suspensions of microcapsules, respectively.

The droplet size distribution curve of the emulsion and particle size distribution curves of microcapsules that were obtained by fitting the experimental data with Gamma equation are shown in Figure 3.

The droplet size plays an important role in the stability of emulsion systems.In addition, the decrease in droplet diameter increasesthe bioavailability of encapsulated compounds [4]. The obtained microcapsules had mean diameters in the range from 4.65 to 6.76 µm (Table 3), that were in accordance with the results of other studies [5,6,7]. The mean diameters did not change significantly, i.e., all of the microcapsules had an uniform size distribution as shown in Figure 3 with low standard deviation, indicating that the type and concentration of cross-linking agent do not affect the particle size. On the other hand, all of the obtained microcapsules have lower particle mean diameters and lesser polydispersity compared to the starting emulsion, as was expected, taking into account that during the drying process small droplets can aspirate and large droplets can burst if the wall is not sufficiently resistant. Based on the results in Figure 3 and Table 3, suspensions of microcapsules without cross-linking agent, as well as suspensions of microcapsules cross-linked with Ch:FA at mass ratio 1:1, had lower particle mean diameters and narrower size distribution, which is probably due to the influence of the properties of their wall. Namely, the wall of these microcapsules has weaker mechanical properties, in order that the larger ones can burst during the spray drying process.In accordance with the previous observations, and for further detail analysis with the SEM and FTIR technique, microcapsules without cross-linking agent and microcapsules cross-linked with Ch:FA and Ch:GA mass ratio 1:2 were selected.

### 2.4. Morphology of Microcapsules

**Figure 4:**
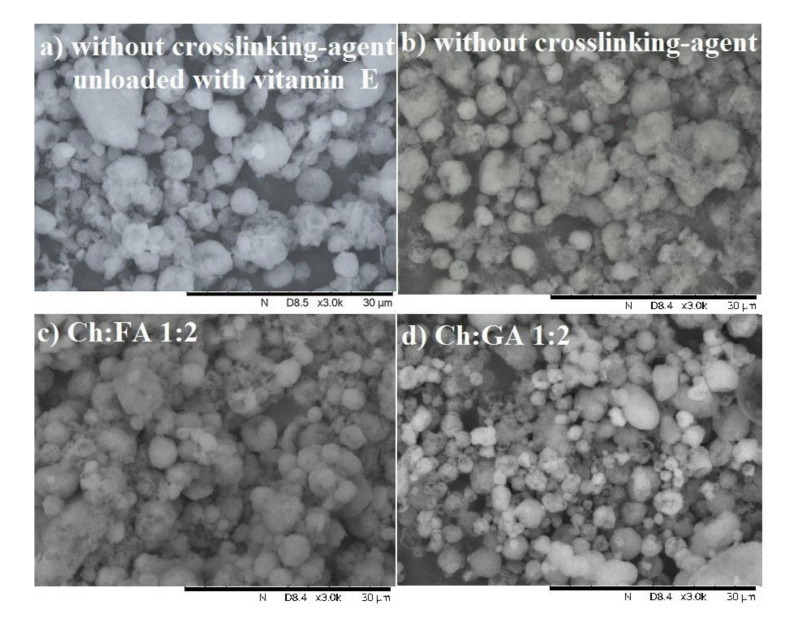
SEM photomicrographs of microcapsules obtained by spray drying of 20% oil-in-water emulsions stabilized with the Ch:SDS complex with or without cross-linking agent (**a**) unloaded and (**b**–**d**) loaded with vitamin E; magnification 3000×.

Figure 4 showed the photomicrographs of optimized microcapsules obtained using SEM.

These microcapsules were successfully prepared, stable through the preparation and spray drying process with an uniform shape and irregular surface. The presence of teeth and rugged surfaces can be attributed to the drying process. There were no significant differences between the microcapsules without cross-linking agent unloaded microcapsules (without vitamin E) and the one loaded with vitamin E (Figure 4a,b). Microcapsules without cross-linking agent and cross-linked with FA (Figure 4a–c) were more adhesive with each other forming aggregates and the number of cracks and sunken areas were present on the microcapsules surface. On the other hand, the microcapsules cross-linked with GA (Figure 4c) were predominantly single, spherical in shape, and with a very slightly wrinkled surface, which indicated their better mechanical characteristics. Similar morphology results were obtained by other authors for different chitosan-based microparticles [7,8,13].However, this was contrary to our previous observations regarding Ch/SLES microcapsules, where microcapsules without cross-linking agent were predominantly single and with the least wrinkled surface [5].

### 2.5. Fourier Transform Infrared Spectroscopy (FTIR) Analysis

**Figure 5:**
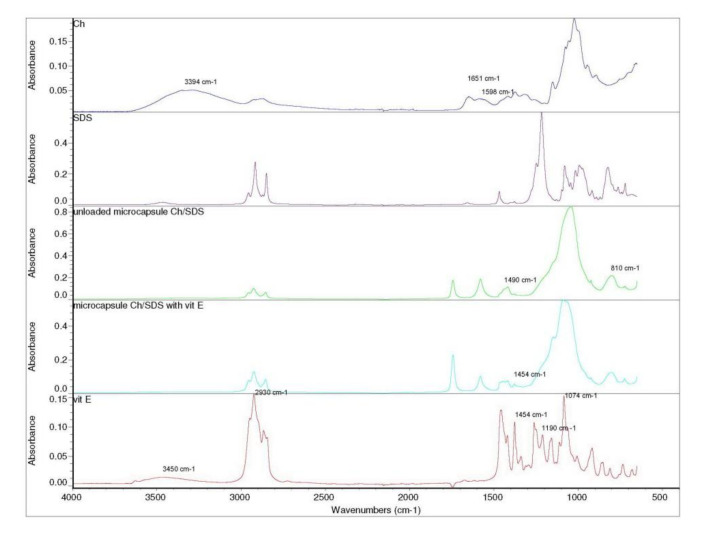
FTIR spectra of chitosan, SDS, vitamin E, unloaded Ch/SDS microcapsules, and Ch/SDS microcapsules loaded with vitamin E.

Figure 5 illustrates FTIR spectrums of chitosan, SDS, vitamin E, unloaded Ch/SDS microcapsules, and Ch/SDS microcapsules loaded with vitamin E.

The spectrum of chitosan showed a characteristic peak at 3394 cm^−1^ corresponding to the stretching vibration of hydroxyl group [39]. The absorption band at 1651 cm^−1^ corresponds to C=O stretching in amide I group and at 1598 cm^−1^ corresponds to N-H bending in amide II group [13]. The spectrums of the microcapsules without cross-linking agent loaded and without vitamin E (Figure 5) show three typical characteristic adsorption peaks. The adsorption bands at 1490 cm^−1^, the asymmetric and symmetric stretching vibration of S-O, and at 810 cm^−1^, the stretching vibration of C-O-S belonging to SDS, but not in the FTIR spectrum of Ch. This indicates that the attachment of the sulfate group of SDS took place on glucosamine groups of Ch [14]. Moreover, the decrease in intensities of adsorption at 810 cm^−1^ in Ch/SDS microcapsules compared to pure SDS, could be indicated on SDS banding to Ch. The FTIR spectrum of vitamin E exhibited characteristic absorption bands, at 1074, 1190, and 2930 cm^−1^ stretching vibration of C=O, C-O, and C-H alkanes group, respectively. The absorption band of vitamin E at 3400–3650 cm^−1^ is attributed to the terminal hydroxyl group and at 1454 cm^−1^ for phenyl skeletal [17,18]. The adsorption bands at 1454 cm^−1^ present at FTIR spectra of vitamin E, but also in the spectra of Ch/SDS microcapsules loaded with vitamin E, but absent in unloaded Ch/SDS microcapsules, could indicate the presence of vitamin E in Ch/SDS microcapsules.

**Figure 6:**
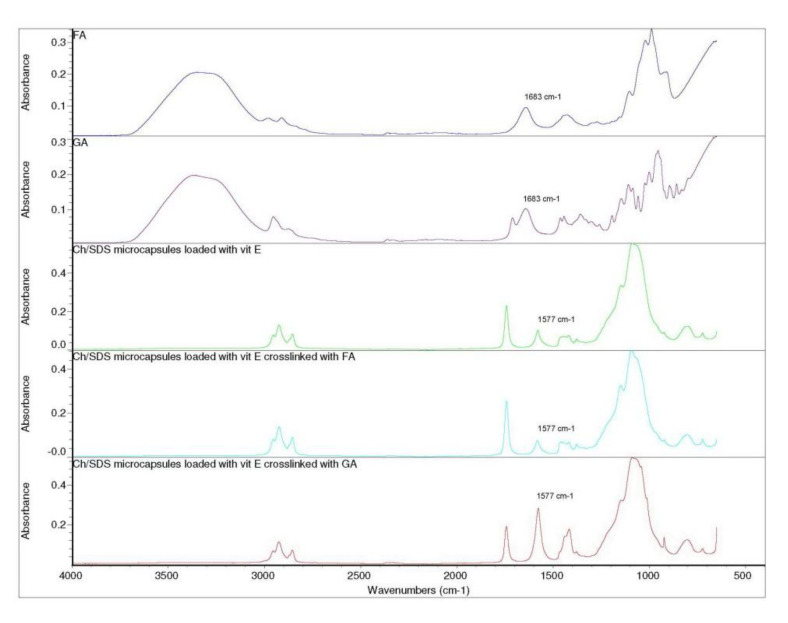
FTIR spectra of FA, GA, Ch/SDS microcapsules loaded with vitamin E without cross-linking agent, as well as cross-linked with FA (mass ratio 1:2) and GA (mass ratio 1:2), respectively.

The spectrum of GA and FA showed a characteristic peak at 1683 cm^−1^ of C=O stretching in the H-bonded pendant aldehyde group. In the case of cross-linked microcapsules with GA and FA, this peak at 1683 cm^−1^ didnot appear, probably due to all of the aldehyde groups that reacted. Moreover, from Figure 6, it can be observed that the absorbance of the peak at 1577 cm^−1^ has increased in microcapsules cross-linked with GA compared to the microcapsules cross-linked with FA and without cross-linking agent. This phenomenon could be attributed to the formation of Schiff’s base between the chitosan and glutaraldehyde [40].

### 2.6. Vitamin E Release Kinetics

Vitamin E (α-tocopherol) is a clear, colorless or yellowish-brown, viscous, oily liquid practically insoluble in water and freely soluble in ethanol [38]. Due to the limited solubility of vitamin E in water, it was not possible to use aqueous buffers as an acceptor medium in this study. In a related study [41], the addition of ethanol to the acceptor medium, in order to enhance the solubility of the released substance in the acceptor medium was recommended. Moreover, in a study by Farid et al. [42], it has been shown that in ethanol/water mixtures the critical micellar concentration of SDS increases with the increasing ethanol concentration. Therefore, the use of ethanol at a concentration of 80% as an acceptor medium precluded the potential risk for solubilization of vitamin E in the acceptor medium, which could affect the detected amount of vitamin E released. Furthermore, Sano et al. [43] have shown that the solubility of chitosan in ethanol/water mixtures decreases linearly with the increasing ethanol concentration, and at a concentration of 50% ethanol the solubility of chitosan was less than 0.1%. Therefore, it can be considered that the acceptor medium used in our study (ethanol 80%) could not significantly affect the integrity and porosity of the microcapsule wall and that the expected mechanism of vitamin E release could be the diffusion from the microcapsule core through the wall composed of chitosan/SDS complex.

**Figure 7:**
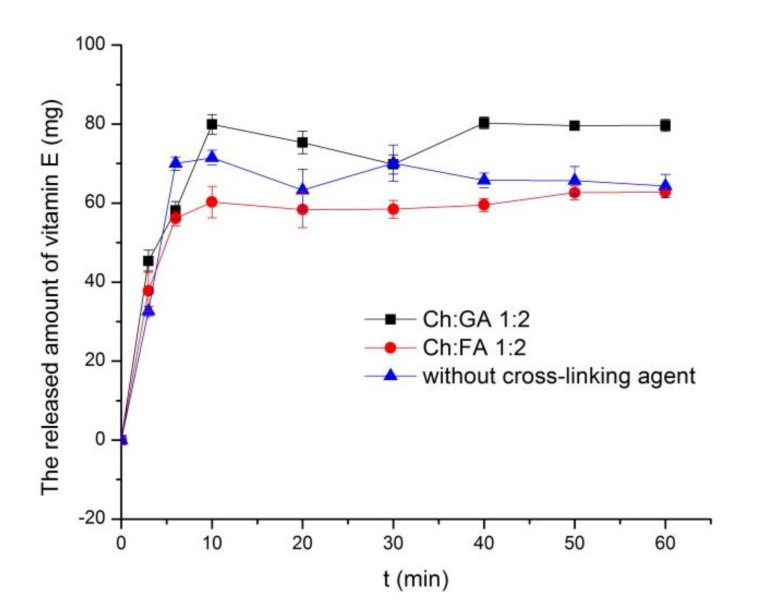
Release profiles of vitamin E from Ch/SDS-based microcapsules.

In order to investigate the properties of the microcapsules in terms of kinetics of vitamin E release under in vitro conditions, the microcapsules without cross-linking agent and the one cross-linked in mass ratio 1:2 were analyzed. The results are shown in Figure 7.

Regarding all of the microcapsules, the release of encapsulated vitamin E lasted for 10 min (Figure 7). Thereafter, a plateau was observed during the next 50 min. The relatively fast release of vitamin E was related with the observation that the cross-linking agents did not establish a sufficiently strong bond with chitosan molecules to reduce the porosity of the wall and thus slow the release of the active substance. Therefore, the release profiles of vitamin E from the microcapsules with and without cross-linking agents were comparable. To estimate the similarity of vitamin E release profiles from the investigated microcapsules, the model-independent analysis was used and the calculated values of *f*_1_ and *f*_2_ are presented in Table 4.

The release profiles can be considered similar for 0 < f_1_ < 15 and/or 50 < f_2_ < 100 [44].The obtained values of f_1_ and f_2_ (Table 4) indicated that the microcapsules containing FA and GA provide similar release profiles. Moreover, the vitamin E release profiles of the microcapsules prepared without crosslinking agent were similar with the one obtained for GA-containing microparticles, while the significant difference was observed in comparison with the vitamin E release profile of FA-containing microcapsules.

Additionally, different mathematical models (Higuchi, first order, zero order, and Korsmeyer-Peppas) were applied to the release profiles, which allowed the attainment of the release kinetic parameters that may provide information regarding the release mechanisms [45,46,47,48]. The values of the correlation coefficient for all of the applied models are shown in Table 5.

The period of time during which the process of vitamin E release took place is in accordance with the time intervals chosen for the analysis. The results in Table 5 indicate that the most appropriate model for this study is the Korsmeyer-Peppas model (r^2^ > 0.9). This model was used to consider the mechanism of release of the encapsulated vitamin E.

It was possible to make an assumption regarding the mechanism of release of the active substance based on the values of the diffusion exponent n (Table 6). The values of this parameter were 0.364 for FA cross-linked microcapsules, 0.490 for GA cross-linked microcapsules, and 0.541 for microcapsules without cross-linking agent. According to the literature, the values of n for microcapsules indicate the following release mechanisms: For n = 0.43, the dominant release mechanism is the Fickian diffusion, while 0.43 < n < 0.85 indicates a diffusion and swelling release mechanism, i.e.,the release is not in full accordance with Fick’s law [49,50]. In the case of microcapsules cross-linked with GA and without a cross-linking agent, the release of vitamin E was based on diffusion through the microcapsule wall. Moreover, the value of n was higher in a sample without a cross-linking agent. Therefore, diffusion was more anomalous than for the sample cross-linked with GA, i.e., probably the polymer swells more when a cross-linking agent was not present. 

Since the surface properties differences between the microcapsules cross-linked with different cross-linking agents were observed, it could be expected that the permeability of the microcapsule wall for diffusion of the encapsulated active substance also depended on the choice of cross-linking agents. The differences between the microcapsules cross-linked with different cross-linking agents were observed. Namely, the diffusion exponent for microcapsules cross-linked with GA was the closest to the value of 0.43, which indicated that there was less swelling compared to the microcapsules without cross-linking agent and that the release of vitamin E was largely controlled by Fick’s diffusion, which is in accordance with the concentration gradient of vitamin E on the outside and inside of the microcapsule wall. In the case of microcapsules cross-linked with FA, the release of vitamin E was less controlled by the diffusion of the carrier (lower value of n). Accordingly, cross-linking Ch with FA created a microcapsule wall of weaker mechanical stability, thus it had a limited effect on the release of vitamin E from the microcapsule core.

The obtained results differed from the results of the previous study of Ch/SLES microcapsules [5,30]. Namely, Ch/SLES microcapsules without cross-linking agent had the lowest value for the encapsulation efficiency and low diffusion exponent (0.234), while for microcapsules cross-linked with GA, the encapsulation efficiency was higher and the vitamin E release was fast with even much lower diffusion exponent (0.045). The Ch/SLES microcapsule wall was too weak to significantly affect the release of E vitamin, thus the release mechanism was described rather as rinsing from the wall surface than as diffusion through the wall of the microcapsule. This finding could be attributed to a different mechanism of interaction between Ch/SLES and Ch/SDS [18,22,23,24], i.e., interactions between Ch and SLES are predominantly hydrophobic, and their coacervate phase isless compact and easy to re-disperse, unlike the Ch/SDScomplex where electrostatic interactions are dominant and the re-dispersibility of their coacervate phase is constrained.

## 3. Materials and Methods

### 3.1. Materials

Ch powder (low molecular weight chitosan, 50,000–190,000 Da, product number: 448869) was obtained from Sigma-Aldrich (Shanghai, China). Ch degree of deacetylation, determined by potentiometric titration according to the procedure described by Yuan et al. [51], was found to be 81.8%. SDS, purity >99%, was purchased from Merck (Darmstadt, Germany). Medium-chain triglycerides (or caprylic/capric triglycerides) (Saboderm TCC, SABO S.p.A, Bergamo, Italy) and vitamin E (α-tocopherol), purified ≥96% (Sigma-Aldrich, Darmstadt, Germany) were used as the oil phase of emulsions. Buffered water was used for the preparation of all solutions, and pH was adjusted at 4.0 using the 0.2 M water solution of acetic acid (Zorka-Pharma, Šabac, Serbia) and 0.2 M water solution of sodium acetate (Centrohem, Stara Pazova, Serbia). GA, 50% solution, purchased from Fisher Chemical (Loughborough, UK) and formaldehyde (FA), 36.5% solution, obtained from Sineks (Belgrde, Serbia), were used as cross-linking agents. Aerosil^®^ 200, fumed silica, was provided from Evonik (Essen, Germany) and ethanol, 96% solution, was purchased from Zorka Pharma (Šabac, Serbia).

### 3.2. Preparation of Polymer and Surfactant Solutions

The stock solution of Ch 0.2% (*w/w*) was prepared by dissolving a given mass of the polymer in the buffered water, at pH 4.0, while stirring and after relaxation at room temperature during 24 h. The pH value of the solution was checked by 827 lab pH-meter (Metrohm, Herisau, Switzerland). The stock solution of SDS 0.4% (*w*/*w*) was prepared by the same procedure. The Ch/SDS mixture at Ch:SDS mass ratio 1:2, suitable for the formation of a stable coacervate [22], was prepared by mixing required volumes of polymer and surfactant stock solutions. This mixture was left for 24 h at room temperature before further use.

### 3.3. Microencapsulation by Spray Drying

Ch/SDS microcapsules were prepared by a complex coacervation method. Initially, the oil-in-water emulsion was prepared. The aqueous phase of emulsion was a solution of Ch/SDS mixture mass ratio 1:2, and the oil phase was a 10% solution of vitamin E in medium-chain triglycerides. The aqueous phase was homogenized for 5 min by Ultra Turrax T25 at 5000 rpm and 30 °C, then the oil phase was gradually added to the aqueous phase during the next first minute of homogenization.Moreover, homogenization was continued for the next 9 min [18,24]. One part of the emulsion was dried immediately after preparation by the spray drying process, i.е., microcapsules were prepared without toxic cross-linking agents, while the other parts were dried after the addition of the cross-linking agent in mass ratio Ch/FA (or Ch/GA) 1:1 and 1:2, and slowly stirring for 2 h at the magnetic stirrer. In order to separate the agglomerated droplets before drying, 2% silica was added to all of the emulsions and stirring at the magnetic stirrer was continued until drying. The Mini Spray Dryer (Büchi 190, Flawil, Switzerland) with a standard 0.7 mm nozzle was used to convert the emulsions into microcapsules powders. The inlet temperature was kept at 160 °C and the outlet at 100 °C. The drying parameters during the process, such as aspiration (0.6 m^3^/min) and feeding (2.2 mL/min) were controlled.

### 3.4. Yield Analysis and Moisture Content Determination

The powders recovered from the equipment were collected and stored in a dark container tightly closed, at 4 °C, before further analysis. The product yield was calculated as the ratio between the mass of the output powders that recovered from the equipment at the end of the process, and the mass of the solid content of the initial solution that was fed to the spray dryer chamber.

The moisture content of Ch/SDS microcapsules was determined gravimetrically by oven-drying at 105 °C up to a constant weight [36]. One gram of powder was used and the moisture was expressed asa percentile value. All of the experiments were conducted in triplicate.

### 3.5. Droplet/Particle Size Distribution Characterization

The droplet size distribution of the emulsions and particle size distribution of the suspensions of microcapsules in water were assessed by the microphotographs, which were taken on an optical microscope Biooptica BEL-3000 (Milano, Italy) at 40× magnification. Microphotographs were analyzed using the BELView software, in accordance with Budinčić et al. [5]. The droplet (particle) mean diameter, expressed as the volume-surface mean value, dvs (μm), and the standard deviation, σ (µm), were calculated from the experimental data, as represented by Equations (1) and (2), respectively:

d_vs_ = ∑n_i_ d_i_^3^/∑n_i_d_i_^2^ (1)

σ = (∑n_i_(d_i_ − d_vs_)^2^/∑n_i_)^1/2^ (2)

where d_i_ is the droplet/particle diameter (µm) and n_i_ is the number of droplets/particles. Droplet/particle size distribution curves were obtained by fitting the experimental data with the Gamma-Equation (3):

Yp= G x^m^ e^(−ax)^ (3)

### 3.6. Scanning Electron Microscopy

The morphology of microcapsules was observed by a scanning electron microscope (SEM), Hitachi TM3030 (Hitachi High-Technologies Corporation, Tokyo, Japan), using an acceleration voltage of 15 kV. The optical microscope was used to observe the re-dispersibility of the powder microcapsules. Microcapsules were in the form of aggregates and, after the addition of water, their re-dispersibility, i.e., the ability to disaggregate was observed.

### 3.7. Fourier Transform Infrared Spectroscopy

FTIR spectra of the Ch, SDS, vitamin E, GA, FA, Ch/SDS microcapsules unloaded and loaded with vitamin E, without and with cross-linking agent, were taken at room temperature using a Nicolet IS10 FTIR spectrophotometer (Thermo Fisher Scientific, USA). All of the spectra were recorded in the spectral range 4000–500 cm^−1^, at a resolution of 16 cm^−1^.

### 3.8. Encapsulation Efficiency Determination

The samples of 0.1 g of microcapsules and 10 mL of 80% ethanol were introduced into the tube and the content was vigorously stirred on the Vortex-Genie 2 mixer (IKA^®^, Germany/Königswinter) for 5 min. After extraction of the encapsulated vitamin E, the samples were filtered (Sartorius filter, 0.1 μm size) and the amount of released vitamin E was determined at 290 nm using a Halo DB-20S UV-VIS spectrophotometer (Dynamica Scientific Ltd., Newport Pagnell, UK) [30]. The encapsulation efficiency [5] was calculated by Equation (4) as follows:

E = m_m_/m_t_ × 100% (4)

where m_m_ is the mass of vitamin E released from 1 g of microcapsules and m_t_ is the mass of vitamin E added during the oil phase emulsification process. All of the experiments were carried out in triplicate.

### 3.9. In Vitro Release Study

The release of vitamin E from precisely measured samples of microcapsules (0.1 g) was performed by the method previously described by Budinčić et al. [5]. Briefly, the release kinetics of vitamin E from the samples of microcapsules was investigated in 80% ethanol under continuous stirring at room temperature. Thereafter, the dissolved vitamin E in supernatant aliquots was analyzed during that time. The content of vitamin E was determined by UV–VIS spectrometry, using the Halo DB-20S UV-VIS spectrophotometer (Dynamica Scientific Ltd., UK), and following an analytical method used and validated in the previous study [32]. All of the experiments were conducted in triplicate.

Finally, the obtained release profiles were analyzed by different mathematical models (zero order, first order, Higuchi, and Korsmeyer-Peppas), which allowed the calculation of vitamin E release kinetic parameters that, in turn, provided some important information regarding the microcapsules release mechanisms [35,36,37].

For the comparison of the obtained vitamin E release profiles, the model-independent approach [44] was used (i.e., calculation of difference (f_1_) and similarity (f_2_) factors) using DDSolver, an Excel Add-In. The corresponding equations are as follows:

f_1_ = Σ_(t=1−n)_ǀR_t_ − T_t_ǀ/Σ_(t=1−n)_R_t_ × 100

f_2_ = 50log{[1 + 1/n·Σ_(t=1−n)_ (R_t_ − T_t_)^2^]^−0.5^ × 100}

where n is the number of samples, R_t_ is the released amount of vitamin E (%) after the time t (reference/sample 1), and T_t_ isthe released amount of the drug (%) after the time t (test/sample 2).

### 3.10. Statistical Analysis

Experimental data were analyzed by the single factor analysis of variance (ANOVA) with the confidence interval of 95%. When pertinent, the means were compared through Tukey’s Honest Significant Difference (HSD) test.

## 4. Conclusions

The formation of Ch/SDS microcapsules loaded with vitamin E, with and without cross-linking agent, was successfully achieved by complex coacervation. All of the formed microcapsules isolated by spray drying were stable, with more or less a spherical and regular shape, and a moisture content of 1.14 to 1.62%, and they were easily re-dispersed in water. The product yields ranged from 11 to 34%, depending on the cross-linking agent used. The addition of cross-linking agents did not affect the mean diameter of emulsion, but after spray drying, all of the obtained microcapsules had a smaller mean diameter and were less polydisperse. The FTIR spectra confirmed that SDS was successfully bonded onto the chitosan surface, as well as the presence of vitamin E in the microcapsules. Microcapsules without the toxic cross-linking agent have good characteristics: Yield (13%), moisture content (1.14%), encapsulation efficiency (126%), mean diameter (5.12 µm), and predominantly uniform shape. The vitamin E release profiles fit with the Korsmeyer-Peppas model. The calculated diffusion exponent indicates a complex release mechanism that, in addition to simple diffusion, involves a swelling microcapsule wall. In Ch/SDS microcapsules without toxic cross-linkers, the swelling component was more pronounced in relation to diffusion, which is more favorable from the aspect of controlling the process of releasing and protection of the encapsulated active substance. Moreover, the complexation of Ch with SDS enabled the formation of a microcapsule wall, which provides better control of vitamin E diffusion from the microcapsule core compared to the microcapsules based on the Ch/SLES coacervate. Small variations in the chemical structure of anionic surfactant (SDS vs. SLES) can lead to significant differences in Ch-based microcapsules properties, especially in terms of kinetics and mechanisms of release of the encapsulated lipophilic active substance.

Keeping in mind the regulatory status of the components of the microcapsule wall (chitosan and SDS) as pharmaceutical excipients, which are approved for use in pharmaceutical preparations for non-parenteral routes of administration [2], the investigated microcapsules loaded with α-tocopherol would be promising for use in solid and liquid pharmaceutical formulations for oral administration (such as capsules, tablets, oral powders, films and oral suspensions, respectively) as well as in liquid and semi-solid formulations for cutaneous applications, such as lotions, gels, creams, and ointments, respectively. In addition, α-tocopherol is an excellent solvent for many poorly soluble drugs [52]. Therefore, the field of application of the designed microcapsules could be expanded by considering their use as carriers of poorly soluble drug substances, which would require further research. Moreover, the investigated microcapsules could be used as an active ingredient in pharmaceutical preparations and dietary supplements for vitamin E replacement, in the form of capsules and tablets. Furthermore, they could be acceptable for use in food of solid, semi-solid, and liquid consistency, as well as in loose powders, as a food additive.

## Figures and Tables

**Table 1:** The moisture content in samples of the chitosan/sodium dodecyl sulfate (Ch/SDS) microcapsules. Different superscript letters (within one column) represent significant differences (*p* < 0.05), as obtained by ANOVA and Tukey’s HSD test.

Sample of Microcapsules	Moisture ± σ (%)
Without cross-linking agent	1.14 ± 0.275^a^
Ch:GA 1:1	1.27 ± 0.156^a^
Ch:GA 1:2	1.58 ± 0.321^a^
Ch:FA 1:1	1.62 ± 0.216^a^
Ch:FA 1:2	1.49 ± 0.159^a^

**Table 2:** The moisture content in samples of the chitosan/sodium dodecyl sulfate (Ch/SDS) microcapsules. Different superscript letters (within one column) represent significant differences (*p* < 0.05), as obtained by ANOVA and Tukey’s HSD test.

Sample of Microcapsules	Encapsulation Efficiency (%)
Without cross-linking agent	126 ± 5.65 ^a^
Ch:GA 1:1	108 ± 3.92 ^b^
Ch:GA 1:2	94 ± 6.14 ^c^
Ch:FA 1:1	104 ± 6.79 ^b^
Ch:FA 1:2	114 ± 2.37 ^a,b^

**Table 3:** Particle size distribution parameters of the emulsion and microcapsules suspensions in water: d_vs_—Mean diameter; σ—Standard deviation. Different superscript letters (within one column) represent significant differences (*p* < 0.05), as obtained by ANOVA and Tukey’s HSD test.

Sample	d_vs_ ± σ (µm)
Emulsion stabilized with Ch/SDS complex	8.18 ± 0.428 ^a^
Suspension of microcapsules without cross-linking agent	5.12 ± 0.372 ^b^
Suspension of microcapsules Ch:FA 1:1	4.65 ± 0.299 ^b^
Suspension of microcapsules Ch:FA 1:2	5.95 ± 0.368 ^c^
Suspension of microcapsules Ch:GA 1:1	6.76 ± 0.386 ^c^
Suspension of microcapsules Ch:GA 1:2	6.23 ± 0.513 ^c^

**Table 4:** The values of difference (f_1_) and similarity (f_2_) factors for the tested pairs of vitamin E release profiles.

Profiles Compared	f_1_	f_2_
Without cross-linking agent vs. Ch:GA 1:2	18.95	43.79
Without cross-linking agent vs. Ch:FA 1:2	9.88	55.34
Ch:GA 1:2 vs. Ch:FA 1:2	20.04	39.76

**Table 5:** Values of the correlation coefficient (r^2^) for the applied mathematical models.

Sample of Microcapsules/Analysis Interval	r^2^			
	Higuchi	First Order	Zero Order	Korsmeyer-Peppas
Without cross-linking agent (3–10 min)	0.7227	0.3944	0.7897	0.9051
Ch:GA 1:2 (3–10 min)	0.8205	0.9902	0.8685	0.9938
Ch:FA 1:2 (3–10 min)	0.8739	0.6010	0.7407	0.9809

**Table 6:** Parameters of vitamin E release kinetics for the Korsmeyer-Peppas model (diffusion rate constant (K) and diffusion exponent (n)).

Sample of Microcapsules	K	n
Without cross-linking agent	22.51	0.541
Ch:GA 1:2	25.36	0.490
Ch:FA 1:2	26.91	0.364

## Data Availability

The data has been presented in main text.
